# Plasma Exchange in Postpartum Hemorrhage (PPH)-Associated Thrombotic Microangiopathy (TMA): A Case Report

**DOI:** 10.7759/cureus.59623

**Published:** 2024-05-04

**Authors:** Asuka Okawa, Masato Yoshihara, Ayako Osafune, Tomokazu Umezu, Hiroaki Kajiyama

**Affiliations:** 1 Department of Obstetrics and Gynecology, Nagoya University Graduate School of Medicine, Nagoya, JPN; 2 Department of Obstetrics and Gynecology, Kariya Toyota General Hospital, Kariya, JPN

**Keywords:** hellp syndrome, postpartum hemorrhage, postpartum atypical hemolytic uremic syndrome, plasma exchange therapy, thrombotic microangiopathy (tma)

## Abstract

Thrombotic microangiopathy (TMA) is a rare yet potentially life-threatening condition. The diagnosis is difficult as there are other conditions presenting with features akin to TMA during the peripartum period such as eclampsia, preeclampsia, hemolysis, elevated liver enzymes, low platelets (HELLP) syndrome, and antiphospholipid syndrome. A 28-year-old woman with no significant past medical history developed TMA following a massive hemorrhage after an emergency cesarean section at 41 weeks of gestation. This case was finally diagnosed as postpartum hemorrhage (PPH)-associated TMA. The patient fully recovered after plasma exchange therapy. We posit the value of accumulating case reports, given that the documentation on the efficacy of plasma exchange in PPH-associated TMA is limited.

## Introduction

Thrombotic microangiopathy (TMA) is characterized by microangiopathic hemolytic anemia because of vascular endothelial damage, destructive thrombocytopenia, and organ damage because of platelet thrombus. TMA is typically associated with thrombotic thrombocytopenic purpura (TTP), atypical hemolytic uremic syndrome (aHUS), Shiga toxin-producing enterohemorrhagic Escherichia coli infection-related uremic syndrome (STEC-HUS). TTP and aHUS require urgent specific treatment such as plasma exchange and complement inhibitors. Additionally, apart from the typical types of TMA, conditions such as preeclampsia, eclampsia, and hemolysis, elevated liver enzymes, low platelets (HELLP) syndrome may present with clinical and laboratory features resembling TMA [1].

Although the research about TMA is progressing, the pathogenesis of TMA induced by severe postpartum hemorrhage (PPH) remains undefined. Herein, we delineate a case of TMA after PPH, with successful disease mediation via plasma exchange.

## Case presentation

A 28-year-old Japanese primigravida, without any significant medical or familial history, underwent an emergency cesarean section at 41 weeks and two days of gestational age for maternal fever because of chorioamnionitis and category Ⅱ fetal heart rate tracing. The intraoperative period was uneventful, and she delivered a female baby of weight 2.6 kg with Apgar scores of 6 and 9 at one and five minutes, respectively. Meconium-stained amniotic fluid was seen. There were no other significant findings.

Five hours after the procedure, she experienced PPH (1.1 L vaginal bleeding). On examination, her body temperature was 38℃, blood pressure was 68/38 mmHg, and pulse of 107/min, with an O2 saturation of 96% on room air. Her hemoglobin was 6.5 g/dL; platelet count was 145×109/L; fibrinogen was 93 mg/dL; PT-INR of 1.52; activated partial thromboplastin time (APTT) was 46.8 seconds; antithrombin Ⅲ was 53%; fibrin-fibrinogen degradation product (FDP) was 80 μg/mL; D-dimer was 60 μg/mL; thrombin-antithrombin complex (TAT) was 240 ng/mL; plasmin-α2 plasmin inhibitor complex (PIC) was 18.69, μg/mL.

**Figure 1:**
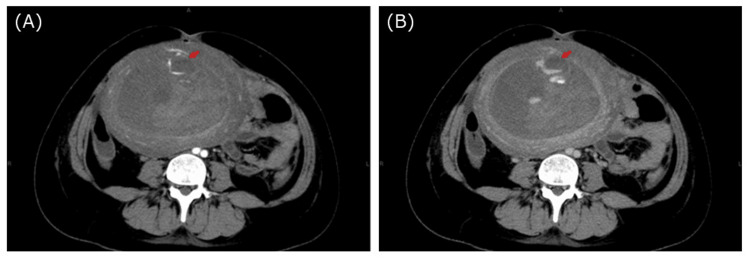
Abdominal contrast-enhanced CT. (A-B): The arrow indicates a hematoma with extravasation.

**Figure 2:**
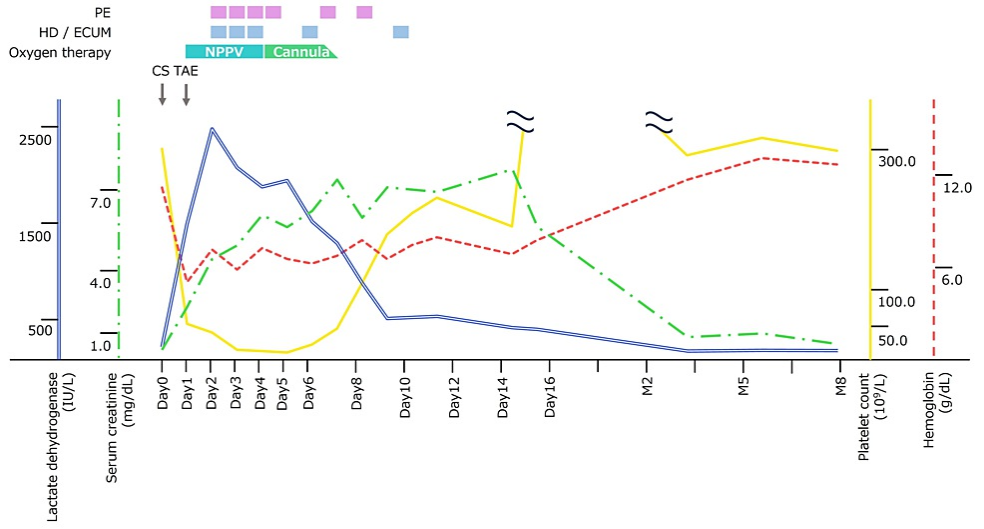
Clinical course: Lactate dehydrogenase, serum creatinine, platelet count, and hemoglobin. PE: plasma exchange; HD: hemodialysis; ECUM: extracorporeal ultrafiltration method; NPPV: noninvasive positive pressure ventilation; CS: cesarean section; TAE: transcatheter arterial embolization.

Contrast-enhanced computed tomography (CT) revealed active uterine artery bleeding (Figure 1). Transcatheter arterial embolization (TAE) was performed with gelatin sponges from both uterine arteries. She required a transfusion of 3.6 L of blood products for stabilization. The patient’s postoperative course is shown in Figure 2.

After TAE, the patient was admitted to the intensive care unit (ICU). She had pulmonary edema, likely the result of a massive blood transfusion, and required respiratory support with noninvasive positive pressure ventilation (NPPV). The fever resolved quickly with ampicillin/sulbactam 3 g every 12 hours. Postoperative day two (POD2), she rapidly developed severe thrombocytopenia (platelet count, 43×109/L) with elevated lactate dehydrogenase (LDH, 2,879 IU/L), undetectable haptoglobin, acute kidney injury (AKI) (creatinine, 3.79 mg/dL), and oliguria (urine output, 0.39 mL/kg/hour). Schistocytes were observed on peripheral blood smear. We conducted laboratory tests to differentiate the causes of TMA such as TTP, STEC-HUS, autoimmune diseases, and other secondary TMA. The results of laboratory tests are shown in Table 1. Genetic tests for aHUS were not available.

**Table 1:** Initial laboratory results at the time of thrombotic microangiopathy onset. WBC: white blood cell; RBC: red blood cell; PT: prothrombin time; INR: international normalized ratio; APTT: activated partial thromboplastin time; FDP: fibrin degradation products; ATⅢ: antithrombin 3; AST: aspartate aminotransferase; ALT: alanine aminotransferase; ALP: alkaline phosphatase; γ₋GTP: gamma-glutamyl transpeptidase; BUN: blood urea nitrogen; CRP: c-reactive protein; LDH: lactate dehydrogenase; ADAMTS13: a disintegrin-like and metalloproteinase with thrombospondin type 1 motif 13; CLβ２GPI: cardiolipin-β2-glicoprotein-I; c1q: complement component 1q; C3: complement3; C4: complement4; CH50: 50% hemolytic unit of complement; C1-INH: C1 inhibitor; TMA: thrombotic microangiopathy; IgG: immunoglobulin-G.

Laboratory test	Results	Reference range
Blood cell count		
WBC	11,700 /μL	3,300-8,600 /µL
RBC	2.18 × 10^6^/μL	3.86-4.92 × 10^6^/µL
Hemoglobin	6.7 g/dL	11.6-14.8 g/dL
Hematocrit	18.7%	35.1-44.4%
Platelet count	43× 10^9 ^L	158-348 × 10^9^/L
Coagulation		
PT activity	78%	70-120%
PT-INR	1.18	0.8-1.2
APTT	34.4 second	27-40 second
Fibrinogen	316 mg/dL	200-400 mg/dL
D-dimer	17.8 µg/mL	<1.0 µg/mL
FDP	22.1 µg/mL	<5.0 µg/mL
ATⅢ	125%	83-118%
Biochemistry		
Sodium	135 mEq/L	138-145 mEq/L
Potassium	4.0 mEq/L	3.6-4.8 mEq/L
Chloride	103 mEq/L	101-108 mEq/L
AST	159 IU/L	13-30 IU/L
ALT	33 IU/L	10-30 IU/L
ALP	75 U/L	106-322 U/L
γ₋GTP	16 U/L	13-64 U/L
Total bilirubin	2.3 mg/dL	0.4-1.2 mg/dL
Direct bilirubin	0.7 mg/dL	0.1-0.6 mg/dL
BUN	37.8 mg/dL	8-20 mg/dL
Creatinine	3.79 mg/dL	0.65-1.07 mg/dL
CRP	28.25 mg/dL	<0.14 mg/dL
LDH	2,879 U/L	120-220 U/L
Haptoglobin	<10 mg/dL	19-170 mg/dL
Additional tests to differentiate the cause of TMA		
ADAMTS13 activity	65%	>10%
ADAMTS13 inhibitor	<0.5 BU/mL	0-0.4 BU/mL
Anticardiolipin antibody (IgG)	<8 U/mL	<10 U/mL
Anti-CLβ2GPI antibody	<1.2 U/mL	<3.5 U/mL
Lupus anticoagulant	Negative	-
C1q	<1.5 U/mL	0-3 U/mL
C3	93 mg/dL	73-138 mg/dL
C4	9 mg/dL	11-31 mg/dL
CH50	44.8 U/mL	25-48 U/mL
C1-INH activity	168%	70-130%

TTP was ruled out because ADAMTS-13 activity was 60% (>10%). There were no gastrointestinal symptoms, and STEC-HUS was also ruled out. HELLP syndrome was an unlikely cause of the TMA because of the prolonged renal dysfunction, even though the mild hepatic enzyme deviations were not typical. We performed plasma exchange for possible TTP at first. The most probable diagnosis was PPH-associated TMA and/or other TMA, which include the possibility of aHUS. When TTP was ruled out, the use of complement inhibitors was considered, but since laboratory data gradually improved, the decision was made to continue plasma exchange.

**Figure 3:**
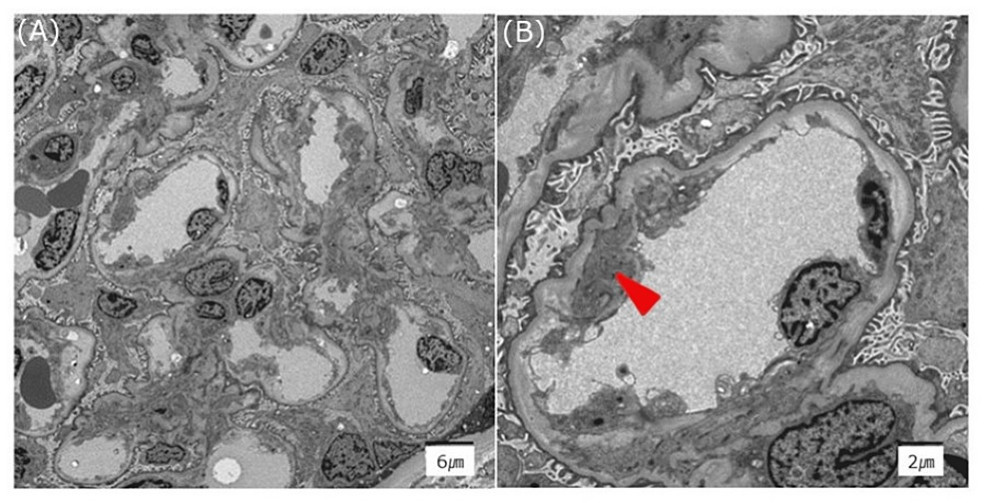
Electron microscopy of the kidney. There is subendothelial expansion of the glomerular basement membrane (A) with partial mesangial interposition (arrow) (B).

The patient underwent six sessions of plasma exchange. Additionally, four sessions of hemodialysis (HD) and one session of extracorporeal ultrafiltration method (ECUM) were performed for AKI and overhydration. A renal biopsy was performed after platelet counts recovered, showing acute tubular necrosis and vascular endothelial damage not contradicting TMA (Figure 3). Her renal function gradually improved, and she was weaned off dialysis. Eight months postpartum, renal function had completely normalized.

## Discussion

We delineate a case of TMA following PPH, managed with plasma exchange. Although research on TTP and some types of aHUS have been growing in recent years and treatment methods have been established, the pathogenesis of many other TMAs remains unclear. Although obstetricians rarely experience cases of TMA, it is known that the vascular endothelium is susceptible during pregnancy and the postpartum period, making them high-risk periods for TMA [1]. The diagnosis of TMA during pregnancy and postpartum can be challenging because the diseases have overlapping features. When thrombocytopenia occurs during pregnancy and postpartum, HELLP syndrome is common in the obstetric field. Extravascular loss, consumption, dilution, and disseminated intravascular coagulation (DIC) are also considered differential diagnoses if there is an association with bleeding. The diagnosis of TTP, aHUS, or other TMAs should also be considered in cases with less typical clinical courses or prolonged symptoms [1-3]. Emerging evidence indicates that aberrations in complement function may play a pivotal role in the pathogenesis of HELLP syndrome [4]. The role of plasma exchange for TMAs including HELLP syndrome has been discussed [5-7].

Recently there is a hypothesis that patients with a genetic predisposition to complement dysregulation may develop complement-mediated TMA triggered by inflammation, infection, and hemorrhagic complication [8,9]. The potential contribution of aHUS to obstetric renal cortical necrosis (RCN) was pointed out [10]. There are reports that some patients developed RCN and dialysis-dependent end-stage renal failure following PPH-induced AKI [11,12]. Supportive care has constituted the cornerstone for PPH-induced AKI, but if there is a possibility that the renal function to be recovered, special interventions such as plasma exchange and complement inhibitors should be considered [12]. There have been reports of plasma exchange for TMA following massive bleeding because of trauma appearing to be similar to PPH-associated TMA [13,14]. The efficacy of plasma exchange should be further investigated.

**Figure 4:**
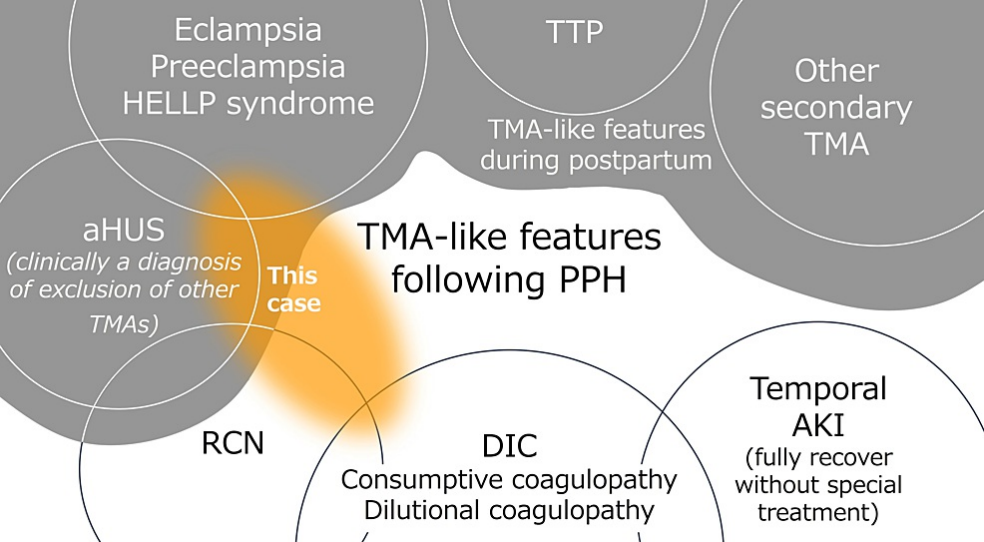
Diseases that present TMA-like features following PPH. TMA, thrombotic microangiopathy; PPH, postpartum hemorrhage; HELLP: hemolysis elevated liver enzymes low platelet syndrome; TTP, thrombotic thrombocytopenic purpura; aHUS: atypical hemolytic uremic syndrome; RCN, renal cortical necrosis; DIC, disseminated intravascular coagulation; AKI, acute kidney injury.

We encountered a case of pregnancy-related TMA, likely associated with PPH (Figure 4). Though renal function is restored following plasma exchange in this instance, the efficacy of plasma exchange in this condition is not certain, and it should not be performed randomly because of the risk of infection and the potentially high medical costs. It is important to recognize that vascular endothelial cells are susceptible to damage during pregnancy. TMA is a rare but serious disease. Early diagnosis is important for early intervention, and obstetricians need to be aware of this. Once the disease has developed, close interdisciplinary collaboration among obstetricians, nephrologists, hematologists, intensivists, and other co-medical workers is imperative for effective TMA management in the perinatal period. Discussion among the entire team, including the patient, is important in determining the course of treatment.

## Conclusions

Current recommendations state that only supportive management is required for TMA associated with PPH, but recently the overlap between PPH-associated TMA and aHUS, and consequently the need for plasma exchange and complement inhibitors has gained attention. The pathogenesis of PPH-associated TMA has not been fully defined, and accumulating data is important. Further research regarding the pathogenesis, long-term prognosis, risk of recurrence, and management strategies for subsequent pregnancies is warranted.
